# Axisymmetric Slow Rotation of Coaxial Soft/Porous Spheres

**DOI:** 10.3390/molecules29153573

**Published:** 2024-07-29

**Authors:** Yu F. Chou, Huan J. Keh

**Affiliations:** Department of Chemical Engineering, National Taiwan University, Taipei 10617, Taiwan; r12524114@ntu.edu.tw

**Keywords:** rotation of soft spheres, creeping flow, hydrodynamic torque, particle interaction

## Abstract

The steady low-Reynolds-number rotation of a chain of coaxial soft spheres (each with an impermeable hard core covered by a permeable porous layer) about the axis in a viscous fluid is analyzed. The particles may be unequally spaced, and may differ in the permeability and inner and outer radii of the porous surface layer as well as angular velocity. By using a method of boundary collocation, the Stokes and Brinkman equations for the external fluid flow and flow within the surface layers, respectively, are solved semi-analytically. The particle interaction effect increases as the relative gap thickness between adjacent particles or their permeability decreases, which can be significant as the gap thickness approaches zero. A particle’s hydrodynamic torque is reduced (its rotation is enhanced) when other particles rotate in the same direction at equivalent or greater angular velocities, but increases (its rotation is hindered) when other particles rotate in the opposite direction at arbitrary angular velocities. For particles with different radii or permeabilities, the particle interaction has a greater effect on smaller or more permeable particles than on larger or less permeable particles. For the rotation of three particles, the presence of the third particle can significantly affect the hydrodynamic torques acting on the other two particles. For the rotation of numerous particles, shielding effects between particles can be substantial. When the permeability of porous layers is low, relative fluid motion is barely felt by the hard cores of the soft particles. The insights gained from this analysis on the effects of interactions among rotating soft particles may be of great importance in many physicochemical applications of colloidal suspensions.

## 1. Introduction

The low-Reynolds-number translation/rotation of small solid particles in a viscous fluid has attracted wide attention from investigators in the fields of biomedical, chemical, civil, environmental, and mechanical engineering as well as physical chemistry. This creeping motion plays an important role in many technological and industrial processes such as sedimentation, centrifugation, coagulation, microfluidics, aerosol technology, rheology of suspensions, as well as electrophoresis and other phoretic motions. The analyses of this topic started from Stokes’ classic work on the translation/rotation of a secluded hard (impermeable to fluid flow) sphere in an incompressible Newtonian fluid [1,2]. Neale et al. [3] and Keh and Chou [4] extended these analyses to the translational and rotational motions, respectively, of a porous (permeable to fluid flow) sphere, like a colloid floc or polymer coil.

A soft (composite) particle of radius b has a hard core of radius a coated with a porous layer of constant thickness b−a [5,6,7,8,9,10]. A biological cell with protein surface attachments [11] and a polystyrene latex with a macromolecular surface layer [12] are examples of a soft particle. To achieve steric stabilization of colloidal dispersions, polymers are intentionally adsorbed onto particles to form permeable layers [13]. The torque exerted on a rotating soft spherical particle by an unbounded fluid of viscosity η is [4]

(1) $$T^{\left(0\right)}=8\pi \eta b^{3}\Omega \left[1+\frac{3}{\lambda^{2}b^{2}}-\frac{3}{\lambda b}\frac{\cosh\left(\lambda b-\lambda a\right)+\lambda \mathrm{asinh}\left(\lambda b-\lambda a\right)}{\sinh\left(\lambda b-\lambda a\right)+\lambda \mathrm{acosh}\left(\lambda b-\lambda a\right)}\right]$$

where $\lambda^{-1}$ and Ω are the flow penetration length (square root of the fluid permeability) in the porous layer and angular velocity, respectively, of the soft sphere (Ω and $T^{\left(0\right)}$ are in opposite directions). In the limit λb→∞, Equation (1) reduces to the Stokes result $T^{\left(0\right)}=8\pi \eta b^{3}\Omega$ for a hard sphere of radius b. In the other limit λb=0, the soft particle has a fully permeable surface layer (without hydrodynamic effect) and Equation (1) becomes $T^{\left(0\right)}=8\pi \eta a^{3}\Omega$. Equation (1) is deduced analytically from the Stokes and Brinkman equations for the external fluid flow and flow within the surface layer of the soft sphere, respectively. The formula for the hydrodynamic drag force and relative velocity of a translational soft sphere corresponding to Equation (1) can also be found in the literature [4,5].

In practical applications, soft particles are not isolated and it is important to understand if the appearance of adjacent particles meaningfully affects the particle movement [14,15,16,17,18,19,20]. Through a method of matched asymptotic expansions combined with a boundary collocation technique, the low-Reynolds-number translation of two soft spheres along their line of centers was examined semi-analytically, and numerical results of hydrodynamic drag forces were calculated for cases of particles with arbitrary sizes and velocities [21]. On the other hand, the axisymmetric translation of multiple soft particles with arbitrary sizes in a concentrated suspension was analyzed by using the boundary collocation method [22]. The particle interaction effect was found to decrease with an increase in the fluid permeability of the surface layers of the particles, be weaker on larger particles than on smaller particles, and be significant as the gap thickness between particles is small.

The purpose of this paper is to analyze the hydrodynamic interaction effect among particles for the slow rotation of a coaxial soft sphere chain about the axis in a concentrated suspension. The particles may differ in size (inner and outer radii, with typical order of micrometers) and fluid permeability of the surface layer as well as angular velocity, and may be unequally spaced along their line of centers. By using the boundary collocation technique, the Stokes and Brinkman equations governing the flow fields of the external fluid and fluid within the surface layers, respectively, are semi-analytically solved and the couples exerted by the fluid on the soft particles are calculated with good convergence. The insights gained from this analysis on the effects of interactions among rotating soft particles at low Reynolds numbers may be of great importance in many physicochemical applications, such as preventing particle flocculation (promoting colloidal stability) or vice versa, optimizing centrifugation processes, and designing micro/nanomotors.

## 2. Analysis

**Figure 1:**
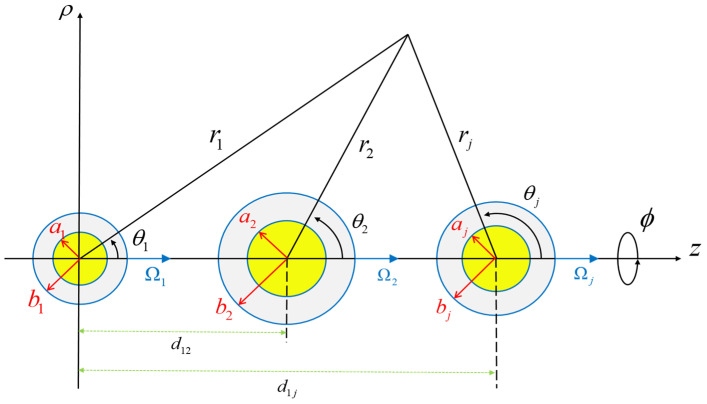
Geometric sketch for the rotation of coaxial soft spherical particles about their axis.

Consider the steady rotation of a straight chain of N soft spheres in an unbounded incompressible Newtonian fluid at rest at infinity about their line of centers (z axis), as shown in Figure 1, under low Reynolds numbers. Here, $\left(r_{i},\theta_{i},\phi \right)$ are the spherical coordinates about the center of particle i and the origin of circular cylindrical coordinates $\left(\rho ,\phi ,z\right)$ is attached to the center of the first particle for convenience. Each soft particle is composed of a spherical hard (impermeable) core and a porous (permeable) surface layer of fixed thickness. The particles may differ in size (inner and outer radii) and permeability of the surface layer and in angular velocity and may be unequally spaced along their line of centers. The objective is to obtain the modification of Equation (1) for each particle’s rotation owing to the presence of other particles.

The outer and inner fluid flows are governed by the Stokes and Brinkman equations, respectively, leading to [23]

(2) $${r_{i}}^{2}\left(\nabla^{2}-\rho^{-2}\right)v_{\phi }=\frac{𝜕}{𝜕r_{i}}\left({r_{i}}^{2}\frac{𝜕v_{\phi }}{𝜕r_{i}}\right)+\frac{𝜕}{𝜕\theta_{i}}\left[\frac{1}{sin\theta_{i}}\frac{𝜕}{𝜕\theta_{i}}\left(v_{\phi }sin\theta_{i}\right)\right]=0$$

(3) $$\frac{𝜕}{𝜕r_{i}}\left({r_{i}}^{2}\frac{𝜕v_{\phi }^{\left(i\right)}}{𝜕r_{i}}\right)+\frac{𝜕}{𝜕\theta_{i}}\left[\frac{1}{sin\theta_{i}}\frac{𝜕}{𝜕\theta_{i}}\left(v_{\phi }^{\left(i\right)}sin\theta_{i}\right)\right]-{\lambda_{i}}^{2}{r_{i}}^{2}\left[v_{\phi }^{\left(i\right)}-\Omega_{i}r_{i}sin\theta_{i}\right]=0$$

where i=1, 2, …, *N*, $v_{\varphi }\left(r_{i},\theta_{i}\right)$ and $v_{\phi }^{\left(i\right)}\left(r_{i},\theta_{i}\right)$ are the φ (only nonzero) components of the fluid velocity distributions outside the particles ($r_{1}\geq b_{1}$, $r_{2}\geq b_{2}$, …, and $r_{N}\geq b_{N}$) and in the permeable surface layer of particle i ($a_{i}\leq r_{i}\leq b_{i}$), respectively, $\lambda_{i}$ is the reciprocal of the flow penetration length in this surface layer, $\Omega_{i}$ is the angular velocity of particle i (which can be either positive or negative), and $b_{i}$ and $a_{i}$ are the radii of the soft particle i and its hard core, respectively. The continuity equations are satisfied, and the dynamic pressure is constant everywhere. In Equation (2), any coordinate system $\left(r_{i},\theta_{i},\phi \right)$ can be used. In Equation (3), $v_{\phi }^{\left(i\right)}$ are the superficial velocities averaged over spaces small regarding the macroscopic dimensions $b_{i}-a_{i}$ but large compared with the pore sizes, and the effective viscosities (embedded in $\lambda_{i}$) are taken to be the same as the bulk fluid viscosity η [5,22].

The boundary conditions for the fluid velocities at the hard core surfaces, at the particle surfaces, and far from the particles are

(4) $$r_{i}=a_{i}:\;v_{\phi }^{\left(i\right)}=\Omega_{i}a_{i}\sin\theta_{i}$$

(5) $$r_{i}=b_{i}:\;v_{\phi }=v_{\phi }^{\left(i\right)},\;\tau_{r_{i}\phi }=\tau_{r_{i}\phi }^{\left(i\right)}$$

(6) $${\left(\rho^{2}+z^{2}\right)}^{1/2}\to \infty :\;v_{\phi }=0$$

where

(7) $$\tau_{r_{i}\phi }=\eta r_{i}\frac{𝜕}{𝜕r_{i}}(\frac{v_{\phi }}{r_{i}}),\;\tau_{r_{i}\phi }^{\left(i\right)}=\eta r_{i}\frac{𝜕}{𝜕r_{i}}(\frac{v_{\phi }^{\left(i\right)}}{r_{i}}),$$

which are the nontrivial components of the viscous stresses.

We can express the fluid velocity distributions in the forms [23,24]

(8) $$v_{\phi }=\sum_{j=1}^{N}\sum_{n=1}^{\infty }A_{jn}{\left(\lambda_{j}r_{j}\right)}^{-n-1}P_{n}^{1}\left(cos\theta_{j}\right)$$

(9) $$v_{\phi }^{\left(i\right)}=\Omega_{i}r_{i}sin\theta_{i}+\sum_{n=1}^{\infty }\left[C_{in}I_{n+\frac{1}{2}}\left(\lambda_{i}r_{i}\right)+D_{in}K_{n+\frac{1}{2}}\left(\lambda_{i}r_{i}\right)\right]{\left(\lambda_{i}r_{i}\right)}^{-\frac{1}{2}}P_{n}^{1}\left(cos\theta_{i}\right)$$

where i=1, 2, …, *N*, $P_{n}^{1}$ is the associated Legendre function of order *n* and degree one, $I_{n}$ and $K_{n}$ are the modified Bessel function of the first and second kinds, respectively, of order n, and $A_{jn}$, $C_{in}$, and $D_{in}$ are the unknown constants to be determined. In Equation (8), the general solutions in N spherical coordinate systems may be superposed since Equation (2) is linear, and boundary condition (6) is immediately satisfied. Substituting Equations (8) and (9) into Equations (4) and (5), we obtain

(10) $$\sum_{n=1}^{\infty }\left[C_{in}I_{n+\frac{1}{2}}\left(\lambda_{i}a_{i}\right)+D_{in}K_{n+\frac{1}{2}}\left(\lambda_{i}a_{i}\right)\right]\;(\lambda_{i}a_{i}{)}^{-\frac{1}{2}}P_{n}^{1}\left(cos\theta_{i}\right)=0$$

$$\sum_{j=1}^{N}\sum_{n=1}^{\infty }A_{jn}[{\left(\lambda_{j}r_{j}\right)}^{-n-1}P_{n}^{1}(cos\theta_{j}){]}_{r_{i}=b_{i}}$$

(11) $$-\sum_{n=1}^{\infty }\left[C_{in}I_{n+\frac{1}{2}}\left(\lambda_{i}b_{i}\right)+D_{in}K_{n+\frac{1}{2}}\left(\lambda_{i}b_{i}\right)\right]{\left(\lambda_{i}b_{i}\right)}^{-\frac{1}{2}}P_{n}^{1}\left(cos\theta_{i}\right)-\Omega_{i}b_{i}sin\theta_{i}=0$$

$$\sum_{j=1}^{N}\sum_{n=1}^{\infty }\left[A_{jn}(n+2)\frac{\lambda_{j}}{\lambda_{i}}{\left(\lambda_{j}r_{j}\right)}^{-n-2}P_{n}^{1}(cos\theta_{j}\right){]}_{r_{i}=b_{i}}+\;\sum_{n=1}^{\infty }\left\{C_{in}[\lambda_{i}b_{i}I_{n-\frac{1}{2}}(\lambda_{i}b_{i})-(n+2)I_{n+\frac{1}{2}}(\lambda_{i}b_{i})\right]$$

(12) $$-D_{in}[\lambda_{i}b_{i}K_{n-\frac{1}{2}}(\lambda_{i}b_{i})+(n+2)K_{n+\frac{1}{2}}(\lambda_{i}b_{i})]\}{\left(\lambda_{i}b_{i}\right)}^{-\frac{3}{2}}P_{n}^{1}(cos\theta_{i})=0$$

where i=1, 2, …, *N*.

In order to express Equations (8), (11) and (12) in a single coordinate system, one can use the conversion formulas between the coordinates $\left(r_{j},\theta_{j}\right)$ and $\left(\rho ,z\right)$ in terms of the distance $d_{ij}$ between the centers of particles i and j,

(13) $$r_{j}={\left[\rho^{2}+{\left(z-d_{1j}\right)}^{2}\right]}^{1/2}$$

(14) $$cos\theta_{j}=\frac{z-d_{1j}}{r_{j}}$$

The satisfaction of the boundary conditions (10)–(12) along the entire inner and outer surfaces of the permeable surface layer of each soft sphere needs the solution of the constants $A_{jn}$, $C_{in}$, and $D_{in}$. The collocation method allows these Equations to be enforced at M points along the longitudinal arc of each spherical surface and the infinite series in Equations (8) and (9) to be truncated after M terms, resulting in 3MN algebraic equations in the truncated form of Equations (10)–(12), which can be numerically solved to yield the 3MN unknown constants $A_{jn}$, $C_{in}$, and $D_{in}$. Details of the boundary collocation scheme are given in a previous article for a straight chain of fluid spheres [25] and slip solid spheres [26] translating along the centerline.

(15) $$T_{i}=8\pi \eta {\lambda_{i}}^{-2}A_{i1},\;i=\;1,\;2,\;\ldots ,\;N$$

The torque acting on the particle i by the fluid can be shown as [23] in which only the lowest-order constant $A_{i1}$ makes contribution. This torque can be expressed as

(16) $$T_{i}=\sum_{j=1}^{N}g_{ij}T_{j}^{\left(0\right)}$$

where $T_{j}^{\left(0\right)}$ is the torque acting on particle j by the fluid in the absence of other particles, given by Equation (1) with $a=a_{j}$, $b=b_{j}$, $\lambda =\lambda_{j}$, and $\Omega =\Omega_{j}$, and the torque correction parameters $g_{ij}$ are functions of the relative sizes, spacings, and flow penetration lengths of the spheres. When all particles are separated by an infinite distance, clearly, $g_{ij}=\delta_{ij}$, a Kronecker delta, which is unity if j=i and zero if j≠i.

When the porous surface layers of all soft particles vanish, the particles reduce to impermeable hard spheres of radius $a_{i}=b_{i}$. In this case, Equations (5), (9), (10) and (12) are trivial, $C_{in}=D_{in}=0$, and merely Equation (11) is required to be solved for the MN constants $A_{jn}$ [27].

When the hard cores of all soft spheres vanish ($a_{i}=0$), the particles become wholly permeable porous spheres of radius $b_{i}$. In this case (the fluid velocity should be finite at the particle centers), Equations (4) and (10) are trivial, $D_{in}=0$, and just Equations (11) and (12) are required to be solved for the 2MN constants $A_{jn}$ and $C_{in}$. The porous spheres become impermeable hard spheres as $\lambda_{i}b_{i}\to \infty$.

## 3. Results and Discussion

First, we present the results obtained from the boundary collocation formulation in the previous Section for the torques exerted by the fluid on two fully porous particles (N=2 and $a_{1}=a_{2}=0$) rotating about their line of centers. Once the constants $A_{jn}$ and $C_{in}$ in Equations (8) and (9) with $D_{in}=0$ for the external and internal fluid velocities are obtained from Equations (11) and (12), Equation (15) is used to calculate the hydrodynamic torques on the particles. In Table 1, numerical solutions of the four torque correction parameters $g_{11}$, $g_{12}$, $g_{21}$, and $g_{22}$ in Equation (16) are given for cases of identical particles ($b_{1}=b_{2}=b$, $\lambda_{1}=\lambda_{2}=\lambda$, $g_{11}=g_{22}$, and $g_{12}=g_{21}$) with numerous values of the particle–particle spacing parameter $\frac{2b}{d_{12}}$ and shielding parameter of the porous layer λb. Typical solutions of these torque correction parameters for cases of porous particles different in either radius or permeability at numerous values of the spacing parameter $\frac{b_{1}+b_{2}}{d_{12}}$ are presented in Table 2. These solutions converge excellently to five digits after the decimal point, and the number of collocation points M=150 along the longitudinal arc of each spherical surface is sufficiently large to achieve this convergence.

In Table 1 and Table 2, for all values of $b_{2}/b_{1}$, $\lambda_{1}b_{1}$, and $\lambda_{2}b_{2}$, the parameters $g_{11}$ and $g_{22}$ are positive and increasing functions of $\frac{b_{1}+b_{2}}{d_{12}}$ from unity at $\frac{b_{1}+b_{2}}{d_{12}}=0$, whereas $g_{12}$ and $g_{21}$ are negative with magnitudes to be also increasing functions of $\frac{b_{1}+b_{2}}{d_{12}}$ but from zero at $\frac{b_{1}+b_{2}}{d_{12}}=0$. These results indicate that the interaction between particles increases as the relative gap thickness $\frac{d_{12}}{b_{1}+b_{2}}$ between particles decreases or the permeability of the porous particles decreases, and this interaction is likely to be significant as $\frac{b_{1}+b_{2}}{d_{12}}\to 1$. The results in Table 1 satisfy the relationship $g_{11}+g_{21}\leq 1$ ($g_{12}+g_{22}\leq 1$), showing that the hydrodynamic torque of one particle is reduced (its rotation is enhanced) by the other particle rotating in the same direction at an equivalent or greater angular velocity, but this torque is increased (its rotation is hindered) by the other particle rotating in the opposite direction at an arbitrary angular velocity. For particles with different radii or permeabilities, the results in Table 2 indicate that the particle interaction has a greater effect on smaller or more permeable particles than on larger or less permeable particles for any given value of $\frac{b_{1}+b_{2}}{d_{12}}$.

For the rotation of three coaxial porous spheres (N=3 and $a_{1}=a_{2}=a_{3}=0$) about their axis, Equation (16) requires nine torque correction parameters to express the torques exerted by the fluid on the particles. For brevity, only the rotation of three particles with the same permeability ($\lambda_{1}=\lambda_{2}=\lambda_{3}=\lambda$) in a symmetric configuration is considered here; that is, the two end particles have identical radii ($b_{3}=b_{1}$) and equal distances from the central particle ($d_{23}=d_{12}=d$). For this symmetric chain, the torque correction parameters are correlated with

(17) $$g_{33}=g_{11},\;g_{32}=g_{12},\;g_{31}=g_{13},\;g_{23}=g_{21}$$

In Table 3, solutions of the torque correction parameters for the rotation of three identical porous particles ($b_{1}=b_{2}=b_{3}=b$) with different values of the spacing parameter $\frac{2b}{d}$ and shielding parameter λb are given. The solutions of the torque correction parameters for the rotation of three porous particles with $\lambda b_{2}=3$ for two typical cases of the relative particle size ($b_{2}/b_{1}$ equal to 2 and 1/2) at different values of the spacing parameter $\frac{b_{1}+b_{2}}{d}$ are presented in Table 4. The particle interactions increase as the permeability of the porous particles decreases and, in general, increase with decreasing $\frac{d}{b_{1}+b_{2}}$, relative spacing between two neighboring particles. The torque correction parameters $g_{11}$, $g_{22}$, and $g_{33}$ are positive and increasing functions of $\frac{b_{1}+b_{2}}{d}$ from unity at $\frac{b_{1}+b_{2}}{d}=0$, whereas the other six torque correction parameters are negative with magnitudes to be in general increasing functions of $\frac{b_{1}+b_{2}}{d}$ but from zero at $\frac{b_{1}+b_{2}}{d}=0$. When the central particle is larger than the end particles, the parameter $g_{31}$ ($=g_{13}$) is not always a monotonical function of $\frac{b_{1}+b_{2}}{d}$. Again, for a given value of $\frac{b_{1}+b_{2}}{d}$, the interaction between particles has a greater effect on the hydrodynamic torques of smaller particles than on those of larger particles.

**Figure 2:**
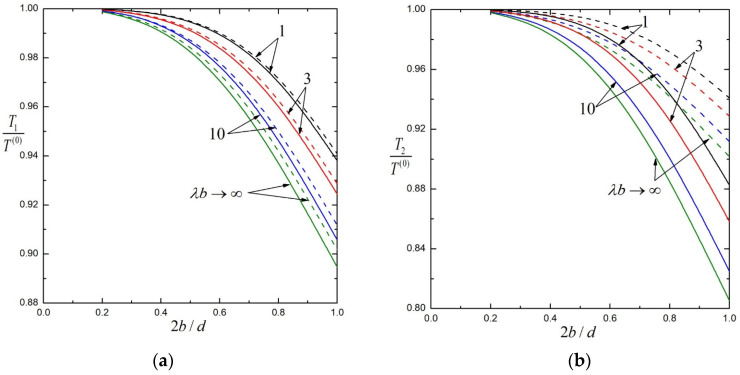
Normalized torques on three identical, equally spaced, porous spheres rotating at equal angular velocities versus the separation parameter $\frac{2b}{d}$ with several values of the shielding parameter λb: (**a**) the first (end) sphere; (**b**) the second (center) sphere. For comparison, the dashed curves are plotted for the torques when only two particles are present.

In Figure 2, the normalized torques $T_{i}^{\;}/T_{\;}^{\left(0\right)}$ exerted by the fluid on the particular case of three identical porous particles ($T_{1}^{\left(0\right)}=T_{2}^{\left(0\right)}=T_{3}^{\left(0\right)}=T_{\;}^{\left(0\right)}$) at equal spacings ($d_{23}=d_{12}=d$) rotating about their line of centers at equal angular velocities ($\Omega_{1}=\Omega_{2}=\Omega_{3}=\Omega$) are plotted against the separation parameter $\frac{2b}{d}$ by solid lines for several values of the shielding parameter λb. The corresponding normalized torques of the first and second particles without the third particle are plotted in the same figure by dashed lines for comparison. All the results in this figure indicate that the torques on particles decrease as λb increases and $\frac{2b}{d}$ increases. Namely, the particle interaction is strongest for a hard (impermeable) sphere chain and weaker for a more permeable sphere chain, and increases as the particles move closer. Apparently, the presence of the third particle reduces the torques of the other two particles for this particular case of three particles rotating in the same direction, and the torque of each particle decreases as the particles move closer. The torque reduction is more significant on the central particle than on the end particles. When the particles come into contact ($\frac{2b}{d}=1$), the existence of the third particle diminishes the torque on the first (end) particle by only about 0.8% for hard (impermeable) particles (λb→∞) and by about 0.3% for porous particles with λb=1, as revealed by Figure 2a (and Table 1 and Table 3). In comparison, as illustrated in Figure 2b (and Table 1 and Table 3), the torque on the second (central) particle is decreased by 10.7% for hard particles and by 6.2% for porous particles with λb=1 when the particles are in contact with each other. Due to configurational symmetry, the torque solutions for two identical porous particles a distance d apart and rotating at equal angular velocities given in Table 1 and Figure 2 are the same as the torque solutions for a single porous particle at a distance d/2 from a free surface plane (without tangential stresses) rotating in the normal direction at the same angular velocity. Here, we do not plot the relevant figure for identical porous particles rotating at equal but opposite angular velocities for brevity.

**Figure 3:**
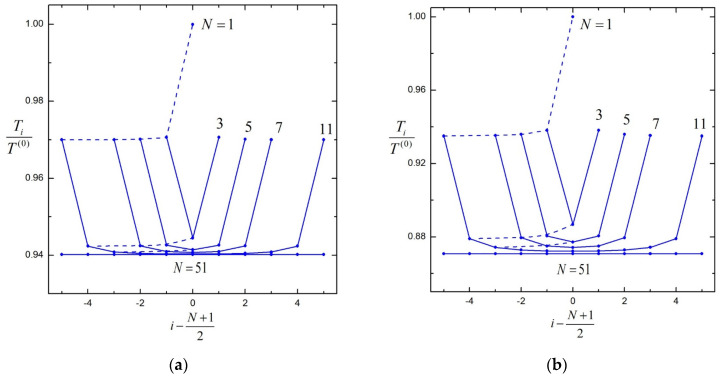
Normalized torques on N identical, equally spaced, porous spheres rotating at equal angular velocities about their axis versus the sphere number i with $\frac{2b}{d}=0.8$: (**a**) λb=1; (**b**) λb=100. Each dashed line connects the particles of the same order (i= 1, 2, or 3) in chains with different numbers (N) of particles.

In Figure 3, the results of the normalized hydrodynamic torques $T_{i}^{\;}/T_{\;}^{\left(0\right)}$ for straight chains of various numbers of N (up to 51) identical porous particles ($a_{i}=0$, $b_{i}=b$, $\lambda_{i}=\lambda$, and $T_{i}^{\left(0\right)}=T_{\;}^{\left(0\right)}$) with identical spacings ($d_{i\left(i+1\right)}=d$) undergoing rotation about their line of centers with identical angular velocities ($\Omega_{i}=\Omega$) are plotted versus the particle number i with $\frac{2b}{d}=0.8$. The torques on the central particles are decreasing functions of the chain length N, showing the shielding effect of particles. When approaching either end of the chain, the relative torques of adjacent particles change quickly, exhibiting strong end effects. For relatively long chains, the torques on the central particles change sluggishly as the chain length increases. In the limit of an infinite chain, the torque on each particle will be the same. The dashed curves in Figure 3, which represent the variation in torque on the *i*th particle as more particles are added to the chain, become flatter as the chain length increases, likewise signifying the shielding effect of particles. Again, the torques on particles in a chain decrease as λb increases, and the particle interaction is weaker for a more permeable sphere chain.

After understanding the particle interaction effects on the axisymmetric rotation of a straight chain of porous spheres, we now examine those of soft spheres. Once the constants $A_{jn}$, $C_{in}$, and $D_{in}$ in Equations (8) and (9) for the fluid velocities are obtained from Equations (10)–(12), Equation (15) is used to calculate the hydrodynamic torques on the particles. In Table 5, numerical solutions of the torque correction parameters $g_{11}$ ($=g_{22}$) and $g_{12}$ ($=g_{21}$) in Equation (16) are given for cases of two identical particles ($a_{1}=a_{2}=a$, $b_{1}=b_{2}=b$, and $\lambda_{1}=\lambda_{2}=\lambda$) with numerous values of the core-to-particle radius ratio a/b, particle–particle spacing parameter $\frac{2b}{d_{12}}$, and shielding parameter of the porous layer λb. Again, $g_{11}+g_{21}\leq 1$, and for all values of a/b and λb, $g_{11}$ is a positive and increasing function of $\frac{2b}{d_{12}}$ from unity at $\frac{2b}{d_{12}}=0$, whereas $g_{12}$ is negative with magnitude to be also an increasing function of $\frac{2b}{d_{12}}$ but from zero at $\frac{2b}{d_{12}}=0$. The particle interaction increases monotonically with increases in the shielding parameter λb and radius ratio a/b (from a wholly porous sphere with $\frac{a}{b}=0$ to a wholly hard sphere with $\frac{a}{b}=1$), keeping other parameters unchanged. For cases where the permeability of porous layers is low (say, λb≥10), the interactions between particles with a modest value of a/b (say, ≤0.8) can be well approached by interactions between corresponding porous particles; namely, the relative fluid motion is barely felt by the hard cores of the soft particles. However, this approximation is unsuccessful for soft spheres with relatively permeable porous layers.

Typical solutions of the torque correction parameters $g_{11}$, $g_{12}$, $g_{21}$, and $g_{22}$ are given for cases of two soft particles different in size with $\frac{a_{1}}{b_{1}}=\frac{a_{2}}{b_{2}}=\frac{1}{2}$ and $\lambda_{2}=\lambda_{1}$ at numerous values of the size ratio $b_{2}/b_{1}$, spacing parameter $\frac{b_{1}+b_{2}}{d_{12}}$, and shielding parameter $\lambda_{1}b_{1}$ are presented in Table 6. Again, the interaction between particles has a greater effect on the hydrodynamic torques of smaller particles than on those of larger particles.

**Figure 4:**
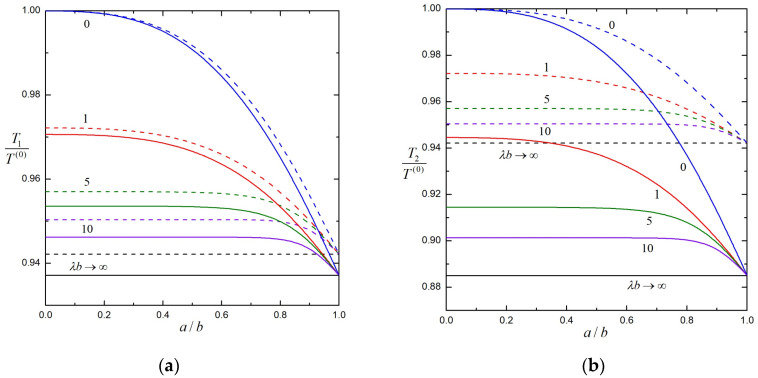
Normalized torques on three identical, equally spaced, soft spheres rotating at equal angular velocities versus the core-to-particle radius ratio a/b with $\frac{2b}{d}=0.8$ and several values of the shielding parameter λb: (**a**) the first (end) sphere; (**b**) the second (center) sphere. For comparison, the dashed curves are plotted for the torques when only two particles are present.

**Figure 5:**
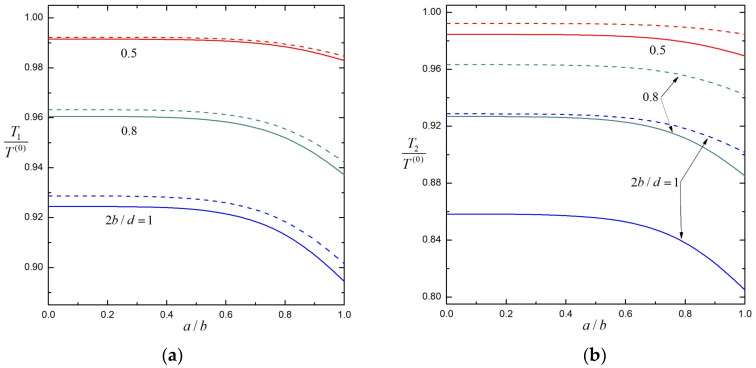
Normalized torques on three identical, equally spaced, soft spheres rotating at equal angular velocities versus the core-to-particle radius ratio a/b with λb=3 and several values of the separation parameter $\frac{2b}{d}$: (**a**) the first (end) sphere; (**b**) the second (center) sphere. For comparison, the dashed curves are plotted for the torques when only two particles are present.

In Figure 4 and Figure 5, the normalized torques $T_{i}^{\;}/T_{\;}^{\left(0\right)}$ exerted by the fluid on three identical soft particles at equal spacings rotating about their line of centers at equal angular velocities are plotted against the core-to-particle radius ratio a/b by solid lines for several values of the separation parameter $\frac{2b}{d}$ and shielding parameter λb. The corresponding normalized torques of the first and second particles without the third particle are plotted in the same figures by dashed lines for comparison. All the results in these figures indicate that, similarly to the case of three porous spheres shown in Figure 2, the torques on particles decrease as λb increases and $\frac{2b}{d}$ increases. The third particle exists to reduce the torques of the other two particles rotating in the same direction, and the torque reduction is more significant on the central particle than on the end particles. Due to symmetry, the torque solutions for two identical soft spheres a distance d apart and rotating at equal angular velocities given in Table 5 as well as Figure 4 and Figure 5 are the same as the torque solutions for a single soft sphere at a distance d/2 from a free surface plane rotating in the normal direction at the same angular velocity.

**Figure 6:**
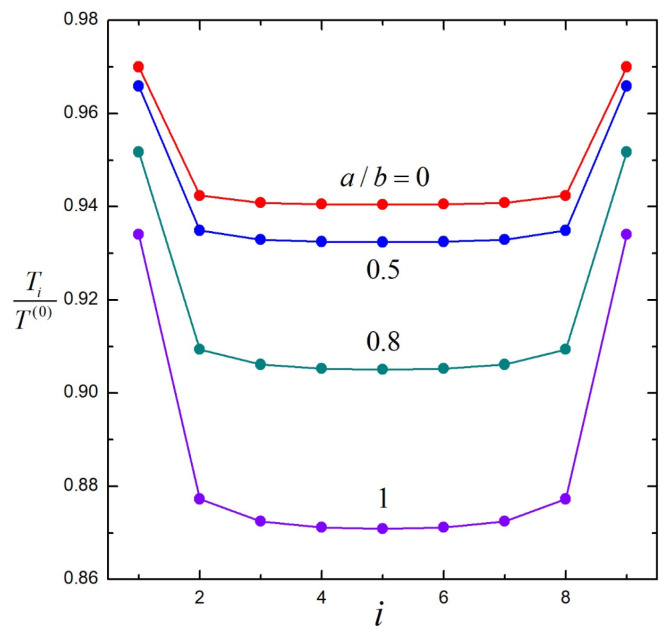
Normalized torques on nine coaxial, identical, equally spaced, soft spheres rotating at equal angular velocities about their axis versus the sphere number i with $\frac{2b}{d}=0.8$, λb=1, and various values of the core-to-particle radius ratio a/b.

Figure 6 is a plot of the normalized torques $T_{i}^{\;}/T_{\;}^{\left(0\right)}$ versus the particle number i for a chain of nine identical and equally spaced soft spheres with λb=1 and $\frac{2a}{d}=0.8$ rotating with equal angular velocities at different values of the core-to-particle radius ratio a/b. These results show that as a/b increases, the torque on each particle in the chain decreases. Particle interactions are strongest for a hard sphere chain and weakest for a porous sphere chain.

## 4. Concluding Remarks

The steady rotation of a chain of coaxial soft spheres about the axis in an incompressible Newtonian fluid is semi-analytically studied at low Reynolds numbers. The particles may differ in the permeability and inner and outer radii of the porous surface layer as well as angular velocity, and may be unequally spaced. By using a method of boundary collocation, the Stokes and Brinkman equations for the fluid flows outside and inside the surface layers, respectively, are solved semi-analytically, and the hydrodynamic torques exerted on the particles are obtained. The particle interaction effect increases as the relative gap thickness between adjacent particles or their permeability decreases, and can be significant as the gap thickness approaches zero. The rotation of a particle is enhanced by other particles rotating in the same direction at equivalent or greater angular velocities, but is hindered by other particles rotating in the opposite direction at arbitrary angular velocities. For particles with different radii or permeabilities, the interaction effect is weaker on larger or less permeable particles than on smaller or more permeable particles. For the rotation of three particles, the presence of the third particle can significantly affect the hydrodynamic torques acting on the other two particles. When the permeability of porous layers is low, the hard cores of the soft particles hardly sense the relative fluid motion.

Although the present article has been confined to the simple axially symmetric rotation of a finite chain of soft spheres, the solution method can be extended to investigate the creeping rotation of any arbitrary three-dimensional assemblage of soft spheres. The results could be a good starting point for future research on other relevant problems, such as the rotation of multiple soft spheres in a non-Newtonian fluid, the rotation of non-spherical soft particles, etc. This analysis is also applicable if the suspended medium is an electrolyte solution and the soft particles are charged. The results obtained from the present study on the interaction effects of rotating soft particles at low Reynolds numbers and their future research implications may hold significance in many physicochemical applications, such as preventing particle flocculation (promoting colloidal stability) or vice versa, optimizing centrifugation processes, and designing micro/nanomotors.

## Figures and Tables

**Table 1:** The torque correction parameters $g_{11}$ ($=g_{22}$) and $g_{12}$ ($=g_{21}$) for the rotation of two identical porous spheres ($a_{1}=a_{2}=0$, $b_{1}=b_{2}=b$, $\lambda_{1}=\lambda_{2}=\lambda$).

	$\frac{2b}{d_{12}}$	λb=1	λb=10	λb=100	λb=600	λb→∞
$g_{11}$	0.2	1.00000	1.00000	1.00000	1.00000	1.00000
	0.3	1.00000	1.00001	1.00001	1.00001	1.00001
	0.4	1.00001	1.00004	1.00007	1.00007	1.00007
	0.5	1.00003	1.00019	1.00028	1.00029	1.00030
	0.6	1.00015	1.00064	1.00093	1.00096	1.00097
	0.7	1.00057	1.00188	1.00263	1.00272	1.00273
	0.8	1.00199	1.00504	1.00677	1.00697	1.00702
	0.9	1.00661	1.01301	1.01674	1.01718	1.01727
	0.99	1.02250	1.03421	1.04216	1.04315	1.04336
	1.0	1.02891	1.04116	1.05012	---	1.05097
$g_{12}$	0.2	−0.00016	−0.00076	−0.00097	−0.00100	−0.00100
	0.3	−0.00069	−0.00260	−0.00329	−0.00336	−0.00338
	0.4	−0.00201	−0.00628	−0.00781	−0.00797	−0.00800
	0.5	−0.00472	−0.01249	−0.01529	−0.01557	−0.01563
	0.6	−0.00954	−0.02200	−0.02648	−0.02694	−0.02704
	0.7	−0.01747	−0.03567	−0.04224	−0.04292	−0.04306
	0.8	−0.02981	−0.05465	−0.06372	−0.06466	−0.06485
	0.9	−0.04880	−0.08118	−0.09339	−0.09467	−0.09494
	0.99	−0.07982	−0.12042	−0.13745	−0.13935	−0.13974
	1.0	−0.08799	−0.12940	−0.14750	---	−0.14943

**Table 2:** The torque correction parameters $g_{11}$, $g_{12}$, $g_{21}$, and $g_{22}$ for the rotation of two porous spheres ($a_{1}=a_{2}=0$) different in radius or permeability.

$\frac{b_{1}+b_{2}}{d_{12}}$	$g_{11}$	$g_{12}$	$g_{21}$	$g_{22}$
$b_{1}=b_{2}=b$, $\lambda_{1}b=1$, $\lambda_{2}b=100$				
0.2	1.00000	−0.00016	−0.00097	1.00000
0.3	1.00000	−0.00069	−0.00329	1.00000
0.4	1.00002	−0.00201	−0.00781	1.00002
0.5	1.00009	−0.00472	−0.01528	1.00009
0.6	1.00036	−0.00955	−0.02646	1.00036
0.7	1.00123	−0.01749	−0.04216	1.00119
0.8	1.00378	−0.02990	−0.06340	1.00348
0.9	1.01123	−0.04923	−0.09234	1.00962
0.99	1.03483	−0.08160	−0.13461	1.02687
1.0	1.04384	−0.09018	−0.14466	1.03290
$\lambda_{1}=\lambda_{2}=\lambda$, $\frac{b_{2}}{b_{1}}=2$, $\lambda b_{1}=3$				
0.2	1.00000	−0.00011	−0.00152	1.00000
0.3	1.00000	−0.00040	−0.00534	1.00000
0.4	1.00002	−0.00099	−0.01323	1.00001
0.5	1.00008	−0.00204	−0.02700	1.00006
0.6	1.00032	−0.00372	−0.04878	1.00021
0.7	1.00112	−0.00622	−0.08117	1.00061
0.8	1.00380	−0.00978	−0.12763	1.00161
0.9	1.01308	−0.01484	−0.19451	1.00399
0.99	1.04988	−0.02251	−0.29702	1.00983
1.0	1.06600	−0.02443	−0.32249	1.01173

**Table 3:** The torque correction parameters for the rotation of three identical porous spheres ($a_{1}=a_{2}=a_{3}=0$, $b_{1}=b_{2}=b_{3}=b$, and $\lambda_{1}=\lambda_{2}=\lambda_{3}=\lambda$) with equal spacings ($d_{23}=d_{12}=d$).

λb	$\frac{2b}{d}$	$g_{22}$	$g_{11}=g_{33}$	$\begin{array}{c} g_{12}=g_{32}\\ =g_{21}=g_{23} \end{array}$	$g_{13}=g_{31}$
1	0.2	1.00000	1.00000	−0.00016	−0.00001
	0.3	1.00000	1.00000	−0.00069	−0.00006
	0.4	1.00001	1.00001	−0.00201	−0.00015
	0.5	1.00006	1.00003	−0.00471	−0.00022
	0.6	1.00029	1.00015	−0.00954	−0.00063
	0.7	1.00114	1.00057	−0.01745	−0.00105
	0.8	1.00398	1.00200	−0.02975	−0.00161
	0.9	1.01321	1.00662	−0.04897	−0.00228
	0.99	1.04494	1.02251	−0.07959	−0.00299
	1.0	1.05777	1.02892	−0.08777	−0.00309
10	0.2	1.00000	1.00000	−0.00076	−0.00009
	0.3	1.00001	1.00001	−0.00260	−0.00031
	0.4	1.00009	1.00004	−0.00627	−0.00072
	0.5	1.00038	1.00019	−0.01247	−0.00136
	0.6	1.00128	1.00065	−0.02195	−0.00223
	0.7	1.00375	1.00189	−0.03554	−0.00329
	0.8	1.01005	1.00506	−0.05437	−0.00447
	0.9	1.02592	1.01304	−0.08067	−0.00568
	0.99	1.06824	1.03426	−0.11964	−0.00676
	1.0	1.08211	1.04120	−0.12858	−0.00687
100	0.2	1.00000	1.00000	−0.00097	−0.00012
	0.3	1.00002	1.00001	−0.00329	−0.00040
	0.4	1.00014	1.00007	−0.00780	−0.00092
	0.5	1.00057	1.00029	−0.01526	−0.00171
	0.6	1.00185	1.00094	−0.02640	−0.00276
	0.7	1.00524	1.00265	−0.04205	−0.00400
	0.8	1.01349	1.00680	−0.06333	−0.00536
	0.9	1.03334	1.01679	−0.09270	−0.00675
	0.99	1.08403	1.04222	−0.13641	−0.00798
	1.0	1.09995	1.05019	−0.14641	−0.00811

**Table 4:** The torque correction parameters for the rotation of three porous spheres ($a_{1}=a_{2}=a_{3}=0$) with $\lambda_{1}=\lambda_{2}=\lambda_{3}=\lambda$, $b_{3}=b_{1}$, $\lambda b_{2}=3$, and $d_{23}=d_{12}=d$.

$b_{1}:b_{2}:b_{3}$	$\frac{b_{1}+b_{2}}{d_{12}}$	$g_{22}$	$g_{11}=g_{33}$	$g_{12}=g_{32}$	$g_{21}=g_{23}$	$g_{13}=g_{31}$
1:2:1	0.2	1.00000	1.00000	−0.00005	−0.00099	−0.00001
	0.3	1.00000	1.00000	−0.00021	−0.00372	−0.00002
	0.4	1.00001	1.00001	−0.00057	−0.00972	−0.00005
	0.5	1.00006	1.00004	−0.00126	−0.02085	−0.00010
	0.6	1.00023	1.00018	−0.00242	−0.03944	−0.00016
	0.7	1.00075	1.00072	−0.00423	−0.06852	−0.00022
	0.8	1.00218	1.00273	−0.00694	−0.11222	−0.00027
	0.9	1.00594	1.01047	−0.01093	−0.17793	−0.00029
	0.99	1.01618	1.04434	−0.01722	−0.28299	−0.00026
	0.999	1.01922	1.05744	−0.01863	−0.30620	−0.00025
	1.0	1.01981	1.06019	−0.01889	−0.31032	−0.00025
2:1:2	0.2	1.00000	1.00000	−0.00152	−0.00011	−0.00018
	0.3	1.00000	1.00000	−0.00534	−0.00040	−0.00062
	0.4	1.00003	1.00002	−0.01321	−0.00099	−0.00150
	0.5	1.00016	1.00007	−0.02690	−0.00204	−0.00298
	0.6	1.00063	1.00024	−0.04846	−0.00370	−0.00519
	0.7	1.00223	1.00069	−0.08025	−0.00614	−0.00827
	0.8	1.00751	1.00179	−0.12528	−0.00959	−0.01232
	0.9	1.02582	1.00437	−0.18913	−0.01442	−0.01747
	0.99	1.09530	1.01040	−0.28510	−0.02150	−0.02321
	0.999	1.12466	1.01211	−0.30702	−0.02318	−0.02386
	1.0	1.12753	1.01229	−0.30931	−0.02335	−0.02394

**Table 5:** The torque correction parameters $g_{11}$ ($=g_{22}$) and $g_{12}$ ($=g_{21}$) for the rotation of two identical soft spheres ($a_{1}=a_{2}=a$, $b_{1}=b_{2}=b$, $\lambda_{1}=\lambda_{2}=\lambda$).

	a/b	$\frac{2b}{d_{12}}$	λb=1	λb=10	λb=100	λb=600
$g_{11}$	0.5	0.2	1.00000	1.00000	1.00000	1.00000
		0.4	1.00001	1.00004	1.00007	1.00007
		0.6	1.00019	1.00064	1.00093	1.00096
		0.8	1.00230	1.00504	1.00677	1.00697
		0.9	1.00724	1.01301	1.01674	1.01718
		0.99	1.02348	1.03421	1.04216	1.04315
		1.0	1.02987	1.04116	1.05012	---
	0.9	0.2	1.00000	1.00000	1.00000	1.00000
		0.4	1.00004	1.00005	1.00007	1.00007
		0.6	1.00064	1.00070	1.00093	1.00096
		0.8	1.00503	1.00543	1.00677	1.00697
		0.9	1.01299	1.01382	1.01674	1.01718
		0.99	1.03411	1.03585	1.04216	1.04315
		1.0	1.04099	1.04292	1.05012	---
$g_{12}$	0.5	0.2	−0.00025	−0.00076	−0.00097	−0.00100
		0.4	−0.00266	−0.00628	−0.00781	−0.00797
		0.6	−0.01141	−0.02200	−0.02648	−0.02694
		0.8	−0.03351	−0.05465	−0.06372	−0.06466
		0.9	−0.05356	−0.08118	−0.09339	−0.09467
		0.99	−0.08551	−0.12042	−0.13745	−0.13935
		1.0	−0.09371	−0.12940	−0.14750	---
	0.9	0.2	−0.00076	−0.00081	−0.00097	−0.00100
		0.4	−0.00628	−0.00665	−0.00781	−0.00797
		0.6	−0.02200	−0.02309	−0.02648	−0.02694
		0.8	−0.05464	−0.05685	−0.06372	−0.06466
		0.9	−0.08116	−0.08412	−0.09339	−0.09467
		0.99	−0.12033	−0.12434	−0.13745	−0.13935
		1.0	−0.12925	−0.13346	−0.14750	---

**Table 6:** The torque correction parameters $g_{11}$, $g_{12}$, $g_{21}$, and $g_{22}$ for the rotation of two soft spheres different in size with $\frac{a_{1}}{b_{1}}=\frac{a_{2}}{b_{2}}=\frac{1}{2}$ and $\lambda_{1}=\lambda_{2}=\lambda$.

$b_{2}/b_{1}$	$\lambda b_{1}$	$\frac{b_{1}+b_{2}}{d_{12}}$	$g_{11}$	$g_{12}$	$g_{21}$	$g_{22}$
2	1	0.2	1.00000	−0.00006	−0.00083	1.00000
		0.4	1.00001	−0.00065	−0.00865	1.00001
		0.6	1.00018	−0.00266	−0.03658	1.00012
		0.8	1.00279	−0.00746	−0.10759	1.00111
		0.9	1.01064	−0.01164	−0.17311	1.00304
		0.99	1.04476	−0.01816	−0.27949	1.00836
		1.0	1.05990	−0.01980	−0.30639	1.01017
	10	0.2	1.00000	−0.00022	−0.00208	1.00000
		0.4	1.00004	−0.00182	−0.01700	1.00003
		0.6	1.00062	−0.00628	−0.05883	1.00042
		0.8	1.00613	−0.01538	−0.14443	1.00274
		0.9	1.01880	−0.02264	−0.21338	1.00627
		0.99	1.06276	−0.03338	−0.31629	1.01416
		1.0	1.07986	−0.03591	−0.34064	1.01647
5	1	0.2	1.00000	−0.00001	−0.00280	1.00000
		0.4	1.00000	−0.00006	−0.02555	1.00000
		0.6	1.00006	−0.00025	−0.09879	1.00003
		0.8	1.00131	−0.00065	−0.27347	1.00019
		0.9	1.00756	−0.00098	−0.43138	1.00047
		0.99	1.06087	−0.00148	−0.69110	1.00112
		1.0	1.09384	−0.00162	−0.76255	1.00133
	10	0.2	1.00000	−0.00003	−0.00440	1.00000
		0.4	1.00001	−0.00022	−0.03562	1.00001
		0.6	1.00022	−0.00076	−0.12179	1.00009
		0.8	1.00320	−0.00182	−0.29449	1.00056
		0.9	1.01417	−0.00264	−0.42955	1.00120
		0.99	1.08505	−0.00388	−0.63510	1.00246
		1.0	1.12507	−0.00422	−0.69222	1.00283

## Data Availability

Data are contained within the article.
